# Improving Time Efficiency in Discharge Documentation Using an AI Copilot Agent: A Prospective Audit in General Adult Psychiatry

**DOI:** 10.1192/bjo.2026.11520

**Published:** 2026-06-30

**Authors:** Catherine Wright

**Affiliations:** Royal Cornhill Hospital, Aberdeen, United Kingdom

## Abstract

**Aims:**

To assess the time difference in creating CDDs and EDDs manually versus using Copilot in general adult psychiatry.

To assess the quality of AI generated CDDs and EDDs.

**Methods:**

Resident doctors will be asked to time themselves while writing a CDD and EDDmanually for a selected patient. They will then use the Copilot agent to generate a CDD and EDD for the same patient and record the time taken. The time difference will be analysed to determine the efficiency gains. Qualitative feedback will also be collected regarding the usability and accuracy of the Copilot-generated documents.

Rationale: The audit was prompted by the auditor’s personal experience with dyslexia and the challenges of managing discharge documentation in a high-pressure clinical environment. The Copilot agent offers a potential solution to reduce documentation time and improve patient flow.

Service areas/teams included: The audit was carried out in Royal Cornhill hospital and included data from general adult psychiatry.

Sample Size: 5 patient cases.

Metrics Recorded:

- Time taken with Copilot vs. without Copilot,
- Number of TrakCare pages referenced,
- Number and nature of mistakes,
- Qualitative comments on errors,
- Minutes saved and percentage time saved,

**Results:**

For CDDs:

- Average time saved: 13 minutes 13 seconds per case.
- Average percentage time saved: 75.3%
- Largest amount of time saved: 17 minutes 34 seconds (≈74.5% reduction).
- Largest percentage of time saved: 76.6%.

For EDDs:

- Average time saved: 26 minutes 22 seconds per case.
- Average percentage time saved: 76.7%.
- Largest amount of time saved: 33 minutes 34 seconds (≈79.9% reduction).
- Largest percentage of time saved: 81.5%.

Error Analysis: Common issues included missing clinical data (e.g., blood results, imaging) due to incomplete inpatient records, medication discrepancies caused by changes not reflected in TrackCare, and errors linked to prior documentation (e.g., suspected perforation). Not all CDDs and EDDs contained all the information about medications due to inaccurate or incomplete record keeping on TrakCare. No errors were due to hallucination by the AI.

Observations: Significant time savings were achieved in most cases. Accuracy depends on completeness of source documentation and integration with TrackCare.

**Conclusion:**

The Copilot agent demonstrates substantial potential to reduce documentation time while maintaining acceptable accuracy. However, integration with real-time clinical data and improved handling of medication updates are essential for reliability.

